# Cystic Fibrosis: A Journey through Time and Hope

**DOI:** 10.3390/ijms25179599

**Published:** 2024-09-04

**Authors:** Pascal Trouvé, Aude Saint Pierre, Claude Férec

**Affiliations:** Univ Brest, Inserm, EFS, UMR 1078, 22 Avenue Camille Desmoulins, F-29200 Brest, France; aude.saintpierre@univ-brest.fr

**Keywords:** cystic fibrosis

## Abstract

Just over thirty years is the span of a generation. It is also the time that has passed since the discovery of the gene responsible for cystic fibrosis. Today, it is safe to say that this discovery has revolutionized our understanding, research perspectives, and management of this disease, which was, thirty years ago, a pediatric condition with a grim prognosis. The aim of this review is to present the advances that science and medicine have brought to our understanding of the pathophysiology of the disease and its management, which in many ways, epitomizes modern molecular genetic research. Since the discovery of the cystic fibrosis transmembrane conductance regulator (CFTR) gene in 1989, modeling the CFTR protein, deciphering its function as an ion channel, and identifying its molecular partners have led to numerous therapeutic advances. The most significant advancement in this field has been the discovery of protein modulators that can target its membrane localization and chloride channel activity. However, further progress is needed to ensure that all patients can benefit from a therapy tailored to their mutations, with the primary challenge being the development of treatments for mutations leading to a complete absence of the protein. The present review delves into the history of the multifaceted world of CF, covering main historical facts, current landscape, clinical management, emerging therapies, patient perspectives, and the importance of ongoing research, bridging science and medicine in the fight against the disease.

## 1. Introduction

In 1989, the year of its discovery, the race for the cystic fibrosis (CF) gene had already begun four years earlier. A significant milestone was reached in 1985, with a genetic linkage study locating a gene associated with the disease on the long arm of chromosome 7 [1,2]. This pivotal moment set the stage for intense scientific competition. European teams, led by Robert Williamson in London, and North American teams, led by Lap Chee Tsui in Toronto embarked on a challenging and competitive journey to identify the gene responsible for CF. The North American consortium ultimately emerged victorious, publishing three seminal articles in the journal *Science* in September 1989, describing successful positional cloning and unveiling a new gene, the CF transmembrane conductance regulator (CFTR) gene responsible for CF [1,3,4]. This discovery was a landmark in the history of medical genetics, transforming our understanding of CF at the molecular level and setting the stage for future research and therapeutic development. It transformed, within a generation, a disease almost exclusively affecting children into a chronic adult condition [5,6,7]. This transformation accompanied a remarkable change in patient management and modified the epidemiological landscape of CF. Indeed, the identification of the CFTR gene not only elucidated the genetic basis of CF but also paved the way for important changes in patients’ care. Prior to this discovery, CF was considered a pediatric disease, and many patients do not reach adulthood. Advancements in medical treatments, due to the increased understanding of the CFTR gene and its mutations, have significantly improved patient outcomes, improving life expectancy and quality of life. Comprehensive care centers, advances in antibiotic therapy, nutritional support, and the development of CFTR modulators are just a few of the innovations that have contributed to this progress. As a result, the median age of survival for individuals with CF has steadily increased. Furthermore, the discovery of the CFTR gene has catalyzed a new era of research focused on understanding the diverse mutations and their effects on CFTR protein function. This has led to the development of personalized medicine approaches, where treatments can be tailored to the specific genetic mutations present in each patient. The introduction of CFTR modulators, which directly target the defective CFTR protein, represents one of the most significant advancements in CF therapy, offering improved lung function and quality of life for many patients.

The present review explores pivotal moments bridging science, medicine and hope for patients over time.

## 2. The History of CF: A Long March towards Hope

### 2.1. From Bewitchment Diagnosis to the Etiology of CF

Whereas medieval folklore predicted death for infants that tasted salty, the earliest accurate medical description of the pancreatic lesion in the case of CF was given in an autopsy of a supposedly bewitched 11-year-old girl. In 1595, the professor of botany and anatomy Pieter Pauw in Leiden (The Netherlands) observed that the meager girl had a swollen, hardened and white pancreas. It was in 1606 that Alonso y de los Ruyzes de Fonteca, professor of medicine at Henares (Spain), wrote that the fingers taste salty after rubbing the forehead of the bewitched child. The modern diagnosis criterion of salty sweat seems to have been seen as a premonition of sickness, emaciation, and death. In the mid-1600s, steatorrhea was reported in some conditions that must have reflected pancreatic insufficiency, another hallmark diagnostic of the disease. Reports from the 19th century and early 20th century associated steatorrhea, meconium complications and abnormal pancreatic lesions with CF. It was K. Landsteiner, the father of the blood group’s definition, who published the first modern description of meconium ileus associated with a defective CF pancreas [8]. Meconium ileus is now considered to be almost pathognomonic of the disease. Fanconi, in 1936, is probably the first to refer to CF as “CF with bronchiectasis” [9]. Later, Andersen, in 1938, used the term “CF of the pancreas” [10]. Because he thought that the cause of CF was a generalized state of thickened mucus, Farber introduced the term *mucoviscidosis* in 1945 to broaden the term used by Andersen [11]. The genetic and the autosomal recessive nature of CF were described in 1946 by Andersen and Hodges [12]. A heat wave in New York resulted in heat prostration among Andersen’s patients at Columbia Hospital. Di Sant’Agnese, working in the same hospital, noticed that salt loss was responsible for the heat prostration, and the primitive folklore of salty-tasting kisses reappeared [13]. He presented this finding to the American Pediatrics Society in 1953 and the sweat test became a common and reliable parameter for differential diagnosis of CF after the improvement of the technique for measuring salt loss by Gibson and Cooke [14]. The sweat test is still considered today as essential for the diagnosis of CF. The seemingly unrelated findings of a thick mucus causing the disease, on the one hand, and an abnormally salty sweat gland producing no significant mucus but being the clearest diagnostic criterion, on the other hand, added more confusion to the understanding of the disease than clarity.

For the next two decades, researchers chased a unifying explanation for the disease’s diverse symptoms but their efforts took many directions, often based on observational data with only tenuous links to establish what was CF.

In 1967, the hypothesis that a circulating humoral factor was responsible for CF gained attention when it was reported that sweat or saliva from patients when applied to the luminal surface of human sweat gland ducts, inhibited active Na^+^ absorption in these epithelia [15]. This was reinforced when a large macromolecule in the tears of patients was found to stimulate excessive mucin release from the urn cell of a marine invertebrate.

A period of scientific wandering ensued, characterized by a multitude of published but non-reproducible and often retracted studies. This was primarily due to the ease of publishing research in this field at the time, without any standardization of studies between different laboratories and without rigorous scientific references and standards. Therefore, the exact nature of CF and a clear definition of the condition still had to be established.

### 2.2. Stuck in the Trail of Mucus

Mucus clearance is the main mechanical defense of the large proximal bronchial and smaller distal bronchiolar airways against the 10^6^ to 10^10^ bacteria and other particulates inhaled per day. The pseudostratified epithelium lining the respiratory airways is composed of three major cell types: basal cells, ciliated cells and non-ciliated secretory cells (goblet cells), which secrete the soluble mucus trapping and keep the microbes away from the host epithelial cell surface. The mucus layer is continuously renewed. Nasal mucus is replaced every 10 min and the lower respiratory tract mucus is transported upward (velocity ≃ 100 μm/s), resulting in its complete turnover within minutes to hours. The main components of mucus are high-molecular-weight mucin glycoproteins (22 different mucin genes have been identified in humans). The major mucins secreted by goblet cells are MUC5AC and MUC5B, whereas MUC1, MUC4, and MUC16 are transmembrane mucins, expressed on the apical surface of all epithelial cells. Glycan structures on the extracellular domains of transmembrane mucins attract water, forming the fluid periciliary layer, essential for ciliary action. A viscoelastic airway mucus gel secreted by goblet cells is located on top of the periciliary layer. MUC5AC is produced by goblet cells in the nasal mucosa and in the tracheobronchial surface epithelium of the lower respiratory tract whereas MUC5B is only expressed in the lower airways. In the case of inflammation, the expression of all mucins is increased but their glycosylation is altered to boost mucosal defense, posing a barrier against bacterial and viral pathogens. However, in CF, respiratory pathogens such as *P. aeruginosa*, *Staphylococcus aureus*, *H. influenzae*, *Streptococcus pneumoniae*, and *Streptococcus pyogenes* have evolved virulence factors to interact with mucin glycans during the course of airway infection [16,17]. In the condition, the almost obligatory link between excessive mucus production and bacterial infections was mentioned as early as 1938 [10] and was further established in the 1950s.

The mucin concentrations are abnormally increased in many diseases such as chronic obstructive pulmonary disease (COPD), non-CF bronchiectasis (NCFB), primary ciliary dyskinesia (PCD), and CF (CF) [18,19,20,21]. Therefore, was the long-held notion that CF was solely a disease of thick and sticky mucus true? While mucus abnormalities, evidenced by mucus plugs and thickening in the pancreas, airways, digestive tract, reproductive system, and salivary glands were indeed observed in various organs of patients, this focus diverted attention from other crucial aspects of the disease. As mentioned above, similar mucus abnormalities occur in other diseases without manifesting the full spectrum of CF symptoms. This raised questions about the specificity of these observations to CF. The chemical composition of mucus in CF, revealed a surprising lack of definitively unique characteristics, challenging the assumption that a distinct mucus composition was a hallmark of CF. This emphasized the need for further exploration beyond just the CFTR protein’s role in mucus production.

A more comprehensive understanding of CF that went beyond the simplistic lens of “viscid mucus” was needed. It was thus proposed that other factors, potentially linked to the function of the CFTR protein, had a significant role in the disease process. This shift in perspective paved the way for new avenues of research and potentially more effective treatment strategies for CF.

### 2.3. Tracing the Ionic Flux

Resurrecting the old notion of salty sweat, the ionic trail was followed.

Initially grappling with the prominent symptom of thick mucus, a deeper understanding of CF lay in the realm of ionic flux, a story enriched by the contributions of dedicated researchers and pivotal discoveries.

Long after the Middle Ages, in the 1930s, Dr. Dorothy H Andersen observed in turn that salty sweat was a constant feature in her patients with CF. This crucial observation, although its significance remained unclear at the time, had hinted at underlying abnormalities in electrolyte concentrations.

By the late 20th century, advancements in technology like improved microscopy and electrophysiological techniques, championed by researchers like Dr. Michael Welsh, opened new avenues [22,23,24]. Scientists began observing disruptions in ion flux across cell membranes, particularly in the chloride channel. This renewed interest in the ionic pathway reignited the search for a deeper understanding of CF. Works performed in the mid-1980s showed that electrolyte transport was abnormal in several CF epithelia. Some of the very first appreciation that CF epithelia were Cl^−^ impermeable came through studies of the sweat gland duct epithelia by Dr. Paul Quinton [25,26]. At about the same time, Drs. Michael Knowles [27,28], Richard Boucher [27,29] and their colleagues highlighted electrolyte transport abnormalities in the airways in CF. When it has been possible to perform similar studies on other organs, related abnormalities have been discovered [24,30,31]. Whereas it was unclear whether CFTR was a Cl^−^ channel or a Cl^−^ channel regulator, it was known that in normal human airway epithelia, it was located in the apical membrane; however, a link with the hydration of mucus remained to be established. Airway epithelia possess the cellular mechanisms for Na^+^ absorption [29,32] which is partly mediated by apical Na^+^ channels that are inhibited by amiloride (Epithelial Sodium Channels, ENaC). In CF, airway epithelia have a two- to threefold greater active Na^+^ transport than normal epithelia [32]. The CF-associated increase in Na^+^ transport was thus thought to alter the quantity and composition of the respiratory tract fluid, contributing to the pathophysiology of CF [29,32]. It was later, at the beginning of the 1990s, that it was demonstrated that CFTR is a Cl^−^ channel [33] regulated by ATP [34], AMPc [35,36,37], PKA and PKC [38]. The increased activity of ENaC in CF cells enhances mucus viscosity and decreases mucociliary clearance, leading to bacterial colonization [39]. Whereas the involvement of amiloride-sensitive Na^+^ channels in CF was observed at the beginning of the 1980s [29,32,40,41], the coupling between ENaC and CFTR was evidenced after the molecular cloning of ENaC [42,43] after the identification of the CFTR gene. However, whether this coupling is direct or indirect remains unclear [37,44,45,46]. Indeed, in the *Xenopus* oocyte expression system and using varying ranges of channel expression levels, no evidence that activated CFTR regulates ENaC was observed when oocyte membrane potential was carefully clamped [47,48]. Nevertheless, inhibiting ENaC could be beneficial to CF patients

### 2.4. Connecting the Dots by Unveiling the CFTR Gene

A pivotal moment arrived in the late 1980s with the groundbreaking work of the Canadian geneticist, Dr. Lap-Chee Tsui. In October 1989, an international collaboration was established under his leadership, creating an International Consortium for the Study of Gene Mutations (CF Gene Mutation Analysis) [1] in which over 100 laboratories around the world collected real-time molecular analysis results and reported over 2000 mutations affecting the gene. The discovery of the CFTR gene was a culmination of years of dedicated research using a technique called positional cloning. A progressive narrowing down of the chromosomal location of the CFTR gene by analyzing genetic linkage between the disease and specific markers on chromosomes was performed. Once the region harboring the gene was identified, DNA sequencing techniques were employed to pinpoint the exact location and sequence of the CFTR gene.

The result obtained was unexpected, and the distribution spectrum of mutated CFTR gene alleles was quite unique, with a very frequent mutation called p.Phe508del (or, according to the official nomenclature of the Human Genome Variation Society: p.Phe508del): a deletion of three nucleotides respecting the reading frame and corresponding to the loss of phenylalanine at position 508 of the protein [49,50,51]. Thanks to the work of P. Farrell and E. Génin (Inserm UMR1078, Brest), we were able to propose that the appearance date of this mutation in European populations was in the Bronze Age (approximately 5000 years BCE) [52]. Today, a selective advantage must account for the high frequency of heterozygotes in our populations, where one in 30 to 35 individuals, depending on the region, carries a mutation in this gene. When considering the genotype of patients, the p.Phe508delmutation is present on the chromosomes of two-thirds of individuals in European populations; only the frequency of a few mutations exceeds the 1% threshold: the G542 mutation (p.Gly542X); the G551D mutation (p.Gly551Asp) such as the 1717-1G>A mutation (c.1585-1G>A); and the W1282X mutation (p.Trp1282stop). We have shown that it is possible to identify almost all mutations in a fairly large population, such as the Breton population [53]. Today, over 2000 variations scattered within the 27 exons of the gene have been reported; these are mostly rare or unique mutations, and the majority of them are causal mutations. The frequency of these mutations varies greatly depending on the geographical or ethnic origin of the patients. For example, the W1282X mutation is the most frequent mutation in the Ashkenazi Jewish population.

Prior to the CFTR gene discovery, diagnosing CF relied on a combination of clinical symptoms and sweat tests. The identification of the gene allowed for the development of more precise and reliable diagnostic tools based on genetic testing. This facilitated earlier and more accurate diagnosis, leading to better patient management and improved outcomes.

The discovery of various CFTR gene mutations revealed a heterogeneous disease landscape. This knowledge paves the way for personalized medicine, where treatment strategies can be tailored to the specific genetic defect of each patient. Indeed, unveiling the CFTR gene fueled the development of novel therapeutic strategies. These include potentiator drugs that enhance the function of partially defective CFTR protein, corrector drugs that address protein folding issues, and gene therapy approaches aimed at correcting the underlying genetic defect. These advancements offer significant hope for improving the quality of life and life expectancy for CF patients.

### 2.5. Autopsy of the CFTR Gene and Its Protein

The past 30 years have seen significant leaps in research, leading to a better understanding of the CFTR gene and protein. It is now known that the CFTR gene is relatively large, spanning 120 kilobases (kb) and comprising 27 exons. It encodes a transmembrane protein of 1480 amino acids. This protein, regulated by cyclic AMPc, consists of two transmembrane domains, two cytoplasmic nucleotide-binding domains (NBD), and a regulatory domain (R). The two NBDs dimerize before channel opening. This conformational change, governed by ATP hydrolysis, regulates chloride conductance [54]. Whereas the CFTR protein (ABCC7) shares many features of ABC transporters, it also possesses specific characteristics such as its long R domain which displays multiple phosphorylation sites for protein kinase A and protein kinase C and its chloride channel function [55,56,57]. Both the amino (N) and carboxyl (C) terminal tails of the protein are oriented towards the cytoplasm and facilitate protein–protein interactions (PPIs) with various proteins, mostly via PDZ interactions [58]. These direct or indirect PPIs with transporters, ion channels, kinases, phosphatases, and cytoskeletal elements that modulate the CFTR’s function also determine its intracellular localization [59,60]. Among the proteins that interact with CFTR, chaperones are particularly important because they influence the protein’s fate based on its folding state [61,62,63]. This folding is often altered in CF, making chaperones potential therapeutic targets against CF. Interestingly, the functional link between CFTR and ENaC has been established, leading to a better understanding of the pathophysiology of CF, particularly regarding the dehydration of periciliary fluids [46,64,65,66]. This allows for the consideration of therapeutic approaches that do not necessarily target the CFTR protein itself.

The structure of the protein is now better understood, and the work of Isabelle Callebaut and Pierre Lehn (Sorbonne University and National Museum of Natural History, Paris) has greatly contributed to it [67,68,69]. They have helped to better understand the structure/function interactions and the impact of nonsense mutations on the protein’s structure [68]. A structure–function analysis of the CFTR protein has further revealed the mechanism by which conformational changes in the channel regulate the Cl^−^ conductance [54]. It has also explained how some mutations causing CF (G551D and L927P) lower the strength of this allosteric pathway, whereas the potentiator ivacaftor enhances it, leading to a better understanding of molecular processes governing the CFTR’s conductance and opening the door for further deciphering of the function of modulators.

**Figure 1:**
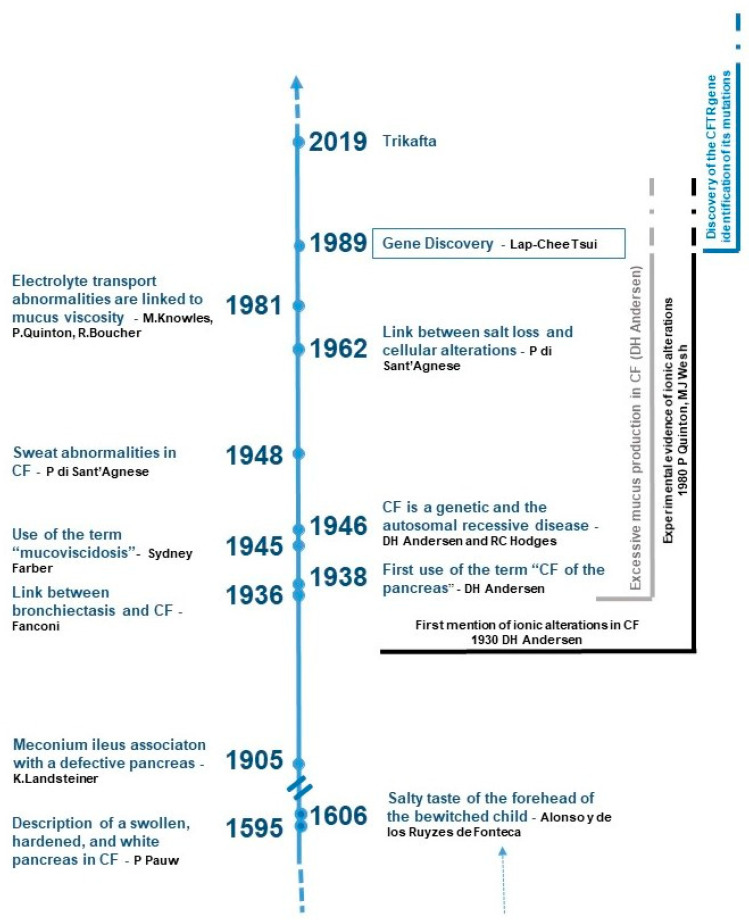
Chronology of the key findings demonstrating that understanding the pathophysiology of cystic fibrosis is a long process. A marked acceleration in the rate of significant discoveries has been observed since the 1930s.

From the early recognition of CF symptoms in the Middle Ages to the groundbreaking discovery of the CFTR gene in 1989 and the characterization of its protein, the journey of scientific exploration has been long and arduous. These milestones have ushered in an era of hope, marked by significant advancements in treatment options and improved patient outcomes (Figure 1). Despite these strides, several aspects of the disease’s pathophysiology and treatment still remain elusive, highlighting the need for ongoing research and innovation.

## 3. Where We Are Today

### 3.1. Mutations, Their Impact on CFTR Function and the Patient’s Phenotype: A Complex Relationship

Whereas the gene is known and the CFTR protein’s function and regulation are better known, the genotype/phenotype relationship in CF remains to be fully elucidated due to the complex interplay between the CFTR gene mutations and various modifying factors. These factors likely include genetic variations, environmental influences, and individual responses that contribute to the diverse clinical manifestations of the disease. Additionally, the presence of over 2000 different CFTR mutations, each potentially affecting the protein function in distinct ways, further complicates the understanding of this relationship. Comprehensive studies integrating genomics, proteomics, and patient data are essential to unravel the precise mechanisms linking genotype to phenotype in CF. Therefore, the years following the gene’s discovery were devoted, probably for the first time in the history of medicine, to an exciting exploration of the relationships between genotype and phenotype. Indeed, many findings have shown the extensive clinical diversity of the disease with, apart from the exocrine pancreatic status, unclear genotype–phenotype relastionships [70,71,72].

The effect of 412 mutations of the CFTR gene has been examined using functional tests, providing evidence of their involvement in the disease: these mutations are described within the North American Consortium coupling molecular data and functional data. A database containing the genotypes of French patients, “CFTR-France”, is also maintained by the Laboratory of Rare Diseases Genetics at the University of Montpellier [73]. It provides precise data on the distribution of mutations in France. This database records all causal and non-causal variations in the CFTR gene sequence and unclassified variants (variants of unknown significance, complex alleles). It also includes all phenotypes associated with mutant alleles that do not correspond to the definition of CF (CFTR-related disorder or CFTR-RD), as well as the results of computational predictions of variant pathogenicity.

The recorded mutations have varying impacts on the function of the CFTR protein. They have been classified into six classes [70]:

Class 1 mutations are nonsense mutations or insertions/deletions or gross deletions leading to a premature stop codon, resulting in the production of an unstable mRNA degraded by NMD (nonsense-mediated decay), thus leading to no synthesis of the CFTR protein and consequently no expression at the apical membrane of epithelial cells.

Class 2 mutations affect protein folding and intracellular trafficking. The emblematic example of these mutations is the P.Phe508delmutation (p.Phe508del), present in at least one copy in 75% of CF patients. The misfolding of the mutated protein disrupts its stability in the endoplasmic reticulum and leads to its early degradation in the proteasome. This mutation is also associated with a defect in chloride channel and a decrease in protein stability. It is thermosensitive: although mutated, at a temperature of 22 °C, the protein is transported to the cell membrane and is functional.

Class 3 mutations affect the control of CFTR channel opening. The protein continues to be expressed on the cell membrane. The G551D mutation (p.Gly551Asp) is the most frequent in this class; it is present in 2 to 4% of patients. It inactivates the opening of the channel, which depends on ATP. These are referred to as gating mutations or “closing mutations”; they are located in the NBD domains at the ATP binding sites.

Class 4 mutations are missense mutations located in the transmembrane domain of the protein. They affect the channel’s conductance.

Class 5 mutations have consequences on the level of messenger RNA (mRNA) expression and, therefore, decrease the amount of CFTR protein available at the cell membrane.

Class 6 mutations affect the protein’s C-terminal domain. They decrease protein stability and increase endocytosis.

Mutations of classes 1, 2, and 3 have a major impact on protein function. They are termed “severe”. In contrast, mutations of classes 4, 5, and 6 have a moderate effect and often allow for residual protein activity. Despite this classification, predicting the clinical severity of the disease based on genotype alone remains challenging because of various modifying factors. Patients with the same CFTR mutation can exhibit a wide range of symptoms and disease severities, underscoring the need for further research to fully elucidate the genotype/phenotype relationship in CF.

Further exploration thus allowed for a “pas de dance” between genotype and disease expression. When present in two copies in a patient, so-called severe mutations are associated with pancreatic insufficiency and early lung involvement starting in childhood. These are common and classic disease presentations, exemplified by patients homozygous for the p.Phe508delmutation. They account for 50% of CF patients in France and in the western part of Europe. Conversely, the combination of a “severe” mutation and a class 4 mutation, for example, often results in preserved exocrine pancreatic function for many years. These combinations are often accompanied by late colonization by *P. aeruginosa*. In these patients, this milder clinical expression is associated with a longer life expectancy, around 50 years today [74]. It is important to keep in mind that patient environmental factors (smoking, socio-economic factors, treatment compliance, etc.), as well as other genetic factors such as modifier genes often involved in immune defense or inflammation, play an important role in the variability of disease expression [75,76].

### 3.2. To Add Complications to Complexity, Here Are the Cftr-Related Diseases

The poor correlation between genotype and phenotype in CF underscores the complexity of CFTR-related disorders, also called borderline forms. CFTR-related disorders are a group of clinical conditions caused by mutations in the CFTR gene, which result in a spectrum of disease manifestations affecting various organs and tissues. These disorders range from the classic presentation of CF to other conditions such as congenital bilateral absence of the vas deferens (CBAVD), chronic pancreatitis, and disseminated bronchiectasis [77,78,79]. They are characterized by varying degrees of CFTR protein dysfunction and exhibit diverse clinical severity, influenced by a combination of genetic, epigenetic, and environmental factors. They often exhibit varying clinical severity, even among individuals with the same CFTR mutations. For example, a mutation that causes severe CF in one patient might result in a milder CFTR-related disorder in another, or they might even remain asymptomatic. This highlights the influence of other genetic, epigenetic, and environmental factors that modify the expression and function of the CFTR protein.

The diverse clinical presentations of CFTR-related disorders illustrate the challenges in predicting disease outcomes based solely on CFTR genotype. This poor genotype–phenotype correlation emphasizes the need for comprehensive approaches that consider the broader genetic and environmental context, alongside CFTR mutation status, to better understand and manage these conditions.

The discovery of CFTR gene mutation involvement in several organ-related diseases was one of the major surprises in the years following its identification. The most striking example was its implication in male infertility due to the absence of the vas deferens. It had long been known that 99% of men with CF were sterile and had excretory azoospermia due to absent vas deferens. A team from Lille (Inserm U16-Biochemistry of Normal and Pathological Proteins, France) was the first to raise the question of CFTR gene responsibility in the most common form of male infertility [80]. Based on a small series of 18 subjects with absent vas deferens followed in a reproductive center, this team reported that 50% of them carried the p.Phe508delmutation. These findings were further complemented by those of Arturo Anguiano et al. (Center for Human Genetics, Boston, MA, USA) [81], who reported that 40% of these infertile men were in fact compound heterozygotes. It now emerges from various meta-analyses that certain variants, such as the 5T allele located upstream of exon 9, or the R117H mutation (p.Arg117His), are over-represented in these patients [82,83].

The responsibility of CFTR gene mutations as a susceptibility factor for the occurrence of chronic pancreatitis in some patients was well documented in 1998 [84]. This genetic origin of chronic pancreatitis was subsequently confirmed by numerous teams [85,86]. Surprisingly, recent work by Michael J. Welsh’s group (University of Iowa) also showed, through multivariate analysis based on a very large series of obligatory heterozygous subjects (parents of an affected child), that pancreatitis and male infertility occurred significantly more often in patients carrying *CFTR* mutations, although these individuals are considered asymptomatic a priori [87].

Among CFTR-related disorders, CF-related diabetes (CFRD) represents a distinct form of diabetes mellitus that affects up to 50% of adults with CF with negative impacts on respiratory function, nutritional status and survival [88].

These studies have thus led to the proposal of the concept of CFTR-related disorders which are associated with dysfunctions of the CFTR protein, but they do not meet the clinical and biological criteria for CF diagnosis [89].

### 3.3. From the Discovery of the CFTR Gene to Systematic Neonatal Screening

In the 1990s, a few pioneering teams, such as Philip M. Farrell’s team at the University of Wisconsin in the United States, or Georges Travert in France, implemented pilot experiments for neonatal screening based on the measurement of immunoreactive trypsin (IRT) levels, three days after birth. This fairly sensitive measurement was not very specific and the implementation of screening at that time faced a principle: that one should not screen for a disease if there was no curative treatment available. It is now well established that screening in the neonatal period improves the nutritional status of children and allows for their rapid management in specialized centers [90]. The discovery of gene mutations led to the proposal of combining the measurement of IRT with the search for common mutations, resulting in a highly effective test in terms of sensitivity and specificity, as well documented by the work of Virginie Scotet in a pilot experiment [91]. With next-generation sequencing, the ability to identify 99% of CFTR gene mutations today allows for a considerable refinement of genetic counseling. This promotes the implementation of family screening for mutation carriers. All of this has led to a decrease in the incidence of the disease, estimated today at 1 patient per 4500 individuals in France, and a change in the epidemiological data of CF [92].

### 3.4. The Changing Epidemiological Data

CF is not a very rare disease among the European population and our descendants in North America. It affects approximately 7000 patients in France, nearly 30,000 patients in the United States, and around 70,000 patients worldwide [93]. This monogenic disease is transmitted in an autosomal recessive manner, meaning both parents must be heterozygous, each carrying one copy of the mutated gene. For these couples, the risk of giving birth to an affected child is 1 in 4 for each pregnancy.

In the 1960s, children with CF rarely lived beyond the age of 10 years old. Today, the median survival age (which corresponds to the age at which 50% of patients born today can expect to survive if mortality conditions and care remain the same) is 46.2 years in the United States, 52.3 in Canada, and approximately 40 years in France. These results reflect the progress made in diagnosis, specialized care, treatment, and the emergence of new therapies [92,94].

For a long time, the incidence of CF was estimated at 1 in 2500 births in the European population. Today, this incidence is lower, at 1 in 3500 births. These data are becoming increasingly precise with the implementation of systematic neonatal screening at birth. In France, thanks to the screening data established in 2002 nationwide, the incidence is 1 in 4700 births with variable local and regional data (1 in 3000 in Brittany and 1 in 7000 in Île-de-France, for example) [95]. The incidence also varies significantly in different populations worldwide, for example, 1 in 17,000 in Africa and 1 in 32,000 in Asia.

The incidence tends to decrease almost everywhere in Europe due to both neonatal screening, which allows early identification of affected children and offers prenatal screening to at-risk couples for subsequent pregnancies, and cascade screening of carriers in affected families. Note also that the diagnosis of CF can follow the detection of a hyperechoic bowel and can be made in utero as part of the ultrasound monitoring of pregnancies [96,97].

The prognosis for children with CF has greatly improved over the past 30 years. One of the most obvious markers of this change is the proportion of adults, now more than 50% of the affected population in many countries (60% in Canada, for example). In France, the proportion of adults has more than doubled in 35 years, from 29.5% in 1984 to 61% in 2018, and this increase is continuous [98]. The European CF Registry predicts that the number of adult patients will reach 75% by 2025 [99]. Today, CF is becoming an adult disease, with the concern of managing this significant transition to adulthood, integrating these young patients into social life, economic life, career choices, and often the desire for parenthood. The number of pregnancies has also increased significantly in recent years. This significant change in the epidemiology of CF is illustrated by recent publications dedicated to “long survivors”, those patients over 40 years old (11% in France). They generally carry mild mutations and are often diagnosed late. Their study and follow-up are important to understand and identify prognostic indicators of long-survival CF patients.

### 3.5. Advancements in CF Treatment: A Beacon of Hope

Understanding the CFTR gene and its associated protein is crucial for patients with CF, and they closely monitor developments in this area. However, what they eagerly await, and rightfully so, are tangible results and new effective medications that will enhance their quality of life, medications that work for everyone, regardless of their specific mutations.

Over the past few decades, the landscape of CF treatment has undergone a remarkable transformation. Researchers, clinicians, and patients have witnessed groundbreaking developments that promise improved quality of life and extended survival. From precision medicine to gene-targeted approaches, these innovations illuminate a path forward, even as we acknowledge the remaining challenges.

### 3.6. CF and Precision Medicine

For hundreds of years, CF was left untreated, and then was it treated empirically. Then came symptomatic treatments. In 1946, Andersen and di Sant’Agnese used penicillin and sulphonamides to treat repeated respiratory infections. Unfortunately, they did not reach their expected success [100]. In order to improve the diagnosis of CF, Paul di Sant’Agnese’s technique for measuring salt loss was improved by Gibson and Cooke [14], resulting in the sweat test that is still used nowadays. In 1957, Barbara Doyle used respiratory therapy called the “English system”, which consisted of chest percussion and contralateral postural drainage aimed to move bronchial secretions [101]. Whereas pancreatic enzyme therapy was introduced in 1872 by Wilhelm Oliver von Leube, it was only in 1900 that this therapy was demonstrated to reduce excess fecal fat and nitrogen loss. This year, Pankreon, the first commercial preparation, appeared, obtained from bovine and porcine pancreatic extracts. Later, in 1958, Douglas N. Crozier gave his patients food rich in saturated fat and high doses of pancreatic enzymes that improved their nutritional status, growth and augmented lengthen survival [102]. Neonatal screening was introduced in the 1980s, following the results of Jeannette Crossley who used a dried blood spot to detect high concentrations of trypsin in patients [103]. The development of this test permitted initiation of treatments early in the disease and made it possible to introduce prophylactic measures against respiratory infections, for test-positive children [90]. Still in the 1980s, the use of nebulized and/or intravenous antibiotics was implemented to prevent and fight against respiratory infections due to *P. aeruginosa* [104,105].

A spectacular breakthrough occurred in 1985 when Magdi Yacoub performed a heart-lung transplant in severely ill patients with a 72% survival rate at 2 years [106].

The 1990s were very fruitful, with treatments becoming more effective and daring as basic research progressed.

The discovery of the *CFTR* gene, the understanding of the function of the CFTR protein and the identification of the molecular anomalies affecting it, opened up new fields of research and new therapeutic avenues.

As early as the 1990s, after demonstrating that transfection of the non-mutated CFTR gene into altered lymphoid cells could restore protein function [107], numerous gene therapy projects were initiated, sparking great enthusiasm in the medical community. Phase I/II trials, based on the use of adenoviruses (or AAV for adeno-associated virus), lentiviruses, and more recently synthetic vectors [108,109] transferring the “drug” gene were undertaken. Research using AAVs showed tolerable transgene tolerance. Unfortunately, this gene therapy resulted in a lack of efficacy, and the results of phase III trials did not meet expectations, likely due to the absence of specific virus receptors on the surface of pulmonary epithelial cells. Today, further research is still needed to improve targeting and transgene expression in epithelial cells, but the hope of eventually using gene therapy remains as illustrated by a recent paper published in *Science* by Sun et al., who combined transient LNP (lipid nano-particles) and CRISPR/Cas9. This approach led to highly efficient progenitor cell correction that provides long-lasting therapeutic effects with a single dose at least in an animal model [110]. Strategies are being explored to specifically correct the mutated portion of the DNA using the CRISPR technique or by directly modifying the mutated nucleotides using deaminases. These strategies aim to correct the mutation not directly in the patient’s body but in stem cells that are then reprogrammed into respiratory basal cells before being administered to the patient to seed the airways. However, several challenges still need to be addressed, including grafting these cells into their submucosal niche and preventing undesirable mutations when modifying the cells.

Other therapeutic strategies have been developed, focusing on correcting mutations belonging to class 1, which results in a total absence of protein or partial protein sequence (nonsense mutations or insertions/deletions prematurely leading to a stop codon). Strategies for correcting stop codons through readthrough have indeed been applied, initially through the action of antibiotics such as gentamicin. However, the toxicity of these antibiotics makes them less usable in therapy, leading to the development of less toxic synthetic products, such as PTC 124 (Ataluren) [111], for which phase III trials have been conducted for CF, but clinical benefits have remained modest. These PTCs either lead to the degradation of mutated transcripts by the cell’s quality control system, nonsense-mediated decay (NMD), or to the elimination of the truncated protein. The first step of these translational therapies involves restricting the NMD system to allow a small number of transcripts to escape degradation and therefore reach the cytoplasm for translation. Indeed, functional rescue of c.3846G>A in nasal cells cultured from a patient was obtained by inhibition of NMD [112]. However, NMD inhibitors are generally very toxic, and their clinical use is delicate. Other “nonsense suppressors” have been evaluated, but their clinical utility has remained limited. Ataluren [111,113,114] would allow the incorporation of amino acids by tricking stop codons (Gln, Lys, and Tyr at UAA and UAG codons; Trp, Arg, and Cys at UGA codons) due to base mismatches. Similarly, ELX-02, a molecule derived from aminoglycosides, showed promising in vitro action on stop codon mutations, but its clinical benefit has not been confirmed. Aminoglycoside antibiotics, such as gentamicin, were among the first molecules identified for their PTC-modifying action [115,116,117].

Research on transcript therapies focuses on mRNA, thus bypassing the delicate step of nuclear translocation of transfected DNA [118,119]. A promising approach currently under significant research involves the design of synthetic tRNAs capable of decoding stop codons [118,120]. These artificial tRNAs compete with termination factors during translation. They allow the introduction of an amino acid into the growing peptide chain, which then continues despite the presence of the stop codon. Produced in sufficient quantities, these proteins can enable the restoration of CFTR channel function at the tissue or organism level. Using anticodon-engineered tRNAs, this could be achieved for G542X, R1162X (c.3484C>T) and W1282X [112]. In vitro, delivering non-mutated mRNA into a mutated cell effectively allows the synthesis of a normal CFTR protein, regardless of the initial mutation carried (mutation-agnostic, with the treatment predicting a good response regardless of the mutation) [121]. These research efforts are highly dynamic, particularly regarding delivery vectors, and the success of mRNA vaccines during the COVID-19 pandemic has marked a turning point in this field. Current strategies rely on chemically modified mRNA (cmRNA) to make them more stable and limit immune reactions due to the detection of exogenous genetic material by the cell. In CF, the first phase II clinical trials have shown the absence of toxicity and good tolerance of these therapies, but without clinical efficacy; the administration frequency to optimize the quantity of CFTR proteins at the membrane is still to be determined.

Significant and highly encouraging progress emerged about a decade ago with the use of small molecules acting specifically on the CFTR protein. These compounds are called modulators. They interact directly with the CFTR protein and increase the CFTR’s channel function (potentiators) or promote its folding and trafficking to the plasma membrane (correctors). Other therapies target proteins that interact with CFTR to increase the amount of CFTR at the membrane (proteostasis modulators and stabilizers) or its function (CFTR activators).

Potentiators form a class of small molecules including compounds capable of interacting with CFTR proteins harboring class 3 mutations, particularly the G551D mutation (p.Gly551Asp), increasing the CFTR channel opening capacity. The flagship of these potentiators is Ivacaftor, whose action was first demonstrated in vitro [122]. Phase II and Phase III trials subsequently showed its effectiveness in patients with the G551D mutation: in these patients, ivacaftor increases forced expiratory volume in one second (FEV1) by approximately 10% after 15 days of treatment, reduces pulmonary exacerbations by 55%, and decreases sweat chloride concentration by 48 mmol. Patients also gain an average of 2.7 kg in weight [123]. Kalideco^®®^, the film-coated form of ivacaftor, was the first modulator to demonstrate efficacy in clinical settings. Its action has been extended to other mutations involved in channel closure, such as S549S or G551S mutations. Other potentiating molecules have been discovered since, and some are undergoing preclinical testing. The main potentiators are presented in Table 1.

Correctors are small molecules that improve the intracellular trafficking of mutated CFTR protein from the endoplasmic reticulum to the apical membrane. During this migration, it undergoes quality control that identifies improperly folded proteins [124]. If this is the case, chaperone proteins take care of them to ensure they are degraded [125]. Numerous compounds with corrector effects were identified by high-throughput screening but they generally had low efficacy [126]. Nevertheless, two different mechanisms of action were initially postulated for compounds acting as correctors [127]. First, these compounds may directly bind to the misfolded protein, improving its defective folding. These correctors are referred to as pharmacological chaperones, to recall the molecular chaperones such as Hsps that promote protein folding [127]. Second, some of the identified correctors were more likely to act through an alternative mechanism of action modulating (directly or indirectly) specific proteins or pathways involved in the CFTR’s folding or degradation. These correctors are referred to as proteostasis regulators. The main correctors are presented in Table 2.

Numerous molecules with corrective activity have been developed to correct the trafficking of mutated CFTR protein. Vertex Pharmaceuticals has developed a promising new compound, VX-809 [128], or lumacaftor, that inserts into a hydrophobic pocket of the TMD1 of CFTR, increasing its stability [129]. It was further proposed to combine it with ivacaftor (this combination has been marketed as Orkambi^®®^) [130,131]. Two phase III studies were conducted to evaluate the efficacy and toxicity of this combination in patients homozygous for the p.Phe508delmutation of the CFTR gene. They showed benefit for patients, with an increase in FEV1 (between 2.6 and 4%), a significant improvement in terms of weight gain, and a reduction in pulmonary exacerbations, without any major toxicity issues. Orkambi^®®^ received marketing authorization for the treatment of patients aged 12 and older. This authorization was later extended to children aged 6 to 11 [132]. These modest results obtained in patients homozygous for the p.Phe508delmutation have led several companies to develop new correctors. The corrector VX-445 (elexacaftor) [133] has been proposed in addition to the already prescribed treatment combining ivacaftor and tezacaftor (VX-661) (formulation called Symdeko^®®^) in patients homozygous for the p.Phe508delmutation [134]. This “cocktail” of three compounds (ivacaftor-tezacaftor-elexacaftor), marketed under the name Trikafta^®®^, which allows for an increase of 11% in FEV1, significantly improves not only patients with one or two copies of the p.Phe508del mutation but also composite heterozygous patients carrying a p.Phe508del allele and another mutation with a and whose CFTR protein has retained residual activity. It is thus envisaged to be able to treat 80% of patients with CF [133,134,135,136]. However, for non-eligible patients to these moderators, other strategies of research are still moving on, for example, transcript therapies. Table 3 provides an overview of the primary therapeutic compounds developed by Vertex Pharmaceuticals. Their respective applications are elaborated upon in Table 4.

**Figure 2:**
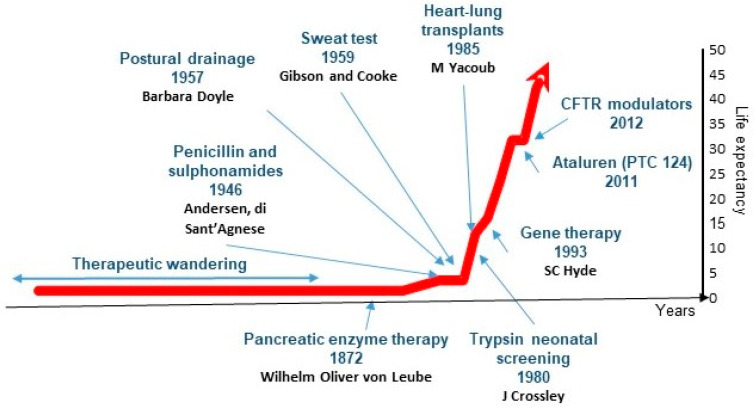
Timeline of the main therapies implemented against cystic fibrosis correlated with the increase in life expectancy. Thanks to new treatments (CFTR modulators), cystic fibrosis is now a chronic adult disease.

The quest for therapies is therefore a long history marked by disappointments but also, fortunately, by successes leading to a dramatic increase in the life expectancy and quality of life of patients (Figure 2). Progress still needs to be made so that all patients can benefit from a treatment that meets their expectations, in relation to their mutations. The era of personalized medicine has begun and, every day, researchers are committed to it.

## 4. Conclusions

There is still a long way to go to better understand the pathogenesis of CF and to discover even more effective molecules and thus improve the life expectancy at birth of children with CF. But what a journey in 30 years! The discovery of the CFTR gene in 1989 stands as a landmark achievement in the field of CF research. It not only unveiled the underlying cause of the disease but also opened avenues for improved diagnosis, development of targeted therapies, and ultimately, the hope for a cure. The constant back-and-forth between fundamental research and clinical research has led to significant advances in just one generation and perfectly illustrates this remarkable cross-fertilization between science and medicine for the benefit of patients.

## Figures and Tables

**Table 1:** Main CFTR potentiators.

Name and Mode of Action	Clinical Trial Phase	Targeted Mutations	Clinical Trial Results
Ivacaftor (Kalydeco^®^)/VX-770 Binds CFTR within the cleft formed by TM 4, 5, and 8 on the TMD2. Increases the probability of CFTR channel opening.	Marketed	G551D, S1251N, and other gating mutations	~10% improvement in FEV1, ~48 mmol/L reduction in sweat chloride concentration
Deutivacaftor/VX-561 Derivates from VX-770 with enhanced in vitro stability and plasma half-life.	Phase 2	Gating mutations	Improved lung function
GLPG-1837 Competitively binds to the same binding site as Ivacaftor. Necessitates a twice daily dosing regimen.	Phase 2	G551D, S1251N	Improved lung function
GLPG-2451 Greater potency than GLPG-1837, supports a once-daily dosing regimen.	Phase 2	Gating mutations	Improved lung function
PTI-808 Structurally resembling Ivacaftor.	Phase 2	F508del and other mutations	Improved lung function, reduced sweat chloride
VX-121/Tezacaftor/Ivacaftor	Phase 3	F508del and other mutations	Not yet published
ABBV-3067	Phase 2	Gating mutations	Not yet published
QBW251	Phase 2	Gating mutations	Not yet published

This table provides an overview of key CFTR potentiators currently in or having completed clinical trials. Many additional potentiators are in preclinical stages and are not shown.

**Table 2:** CFTR correctors.

Name	Clinical Trial Phase	Clinical Trial Results
Lumacaftor (Orkambi^®®^)/VX-809	Marketed	~4–6% improvement in FEV1, ~30–40 mmol/L reduction in sweat chloride concentration1
Tezacaftor (Symdeko^®®^)/VX-661	Marketed	~6–8% improvement in FEV1, ~40 mmol/L reduction in sweat chloride concentration2
Elexacaftor (Trikafta^®®^/Kaftrio^®®^)/VX-445	Marketed	~14% improvement in FEV1, ~45–50 mmol/L reduction in sweat chloride concentration3
ABBV-2222	Phase 2	Not yet published
GLPG-2222	Phase 2	Not yet published
PTI-801	Phase 2	Not yet published

This table provides an overview of key CFTR correctors currently in or having completed clinical trials. Many additional potentiators are in preclinical stages and are not shown.

**Table 3:** Main Vertex products.

Molecule Name	EU Market Name (Year)	CF Mutations
Ivacaftor	Kalydeco 2014	Classes III and IV, gating and conduction mutations, residual function mutations G551D, S549N, G1244E, G178R, S1251N, G551S, G1349D, S1255P, R117H, E56K, K1060T, P67L, E193K, A1067T, R74W, L206W, G1069R, D110E, R347H, D579G, R1070Q, D1270N, D110H, R352Q, S945L, R1070W, R117C, A455E, S977F, F1074L, F1052V, D115H; 3849+10 kb C>T, 2789+5G>A, 3273-26A>G, 711+3A>G, E831X
Lumacaftor/Ivacaftor	Orkambi 2018	Class II, p. Phe508del homozygous
Tezacaftor/Ivacaftor	Symkevi 2018	Two copies of P.Phe508del One copy of P.Phe508del in association with E56K, K1060T, P67L, E193K, A1067T, R74W, L206W, D110E, D110H, R347H, D579G, R1070Q, D1270N, R352Q, S945L, R1070W, R117C, A455E, S977F, F1074L, F1052V, D1152H, 3849+10 kb C>T, 2789+5G>A, 327326A>G, 711+3A>G
Elexacaftor/Tezacaftor/ Ivacaftor	Kaftrio 2020	Class II, at least one copy of P.Phe508del mutation and one copy with residual function mutation

Summary of pivotal Vertex Pharmaceuticals molecules, their European market introduction dates, and the principal cystic fibrosis transmembrane conductance regulator (CFTR) mutations they are designed to treat.

**Table 4:** CFTR modulators currently prescribed to patients.

Name	% Increase in FEV1	Sweat Chloride Concentration	Age	Targeted Mutations
Kalydeco^®®^ (Ivacaftor)	~10%	Reduction of ~48 mmol/L	6 months and older	G551D, S1251N, and other gating mutations
Orkambi^®®^ (Lumacaftor/Ivacaftor)	~4–6%	Reduction of ~30–40 mmol/L	2 years and older	p.Phe508del homozygous
Symdeko^®®^ (Tezacaftor/Ivacaftor)	~6–8%	Reduction of ~40 mmol/L	6 years and older	p.Phe508del homozygous or one F508del and one residual function mutation
Trikafta^®®^/Kaftrio^®®^ (Elexacaftor/Tezacaftor/Ivacaftor)	~14%	Reduction of ~45–50 mmol/L	6 years and older	At least one p.Phe508del mutation

Overview of major CFTR modulators, including their name, percentage increase in forced expiratory volume in one second (FEV1), sweat chloride concentration, patient age, and targeted cystic fibrosis transmembrane conductance regulator (CFTR) mutations.
